# Cure for Sleep-Walking

**Published:** 1872-10

**Authors:** 


### Cure for Sleep-walking

This troublesome habit often resists all the common-place plans to break it up. When such is the case, Dr. Pellizzo, in the *Abeile Medicalle*, recommens the use of bromide of potassium. We tried it in the case of a girl who would get up in her sleep, walk about her room, eat, etc. About 30 grains daily (morning and evening) effectually cured. It is also of good service in the agitated and restless sleep of good children.

